# Automated Detection of Lumbosacral Transitional Vertebrae on Plain Lumbar Radiographs Using a Deep Learning Model

**DOI:** 10.3390/jcm14217671

**Published:** 2025-10-29

**Authors:** Donghyuk Kwak, Du Hyun Ro, Dong-Ho Kang

**Affiliations:** 1 College of Medicine, Seoul National University, Seoul 03080, Republic of Korea; jadeking0308@snu.ac.kr (D.K.); duhyunro@gmail.com (D.H.R.); 2 Department of Orthopedic Surgery, Seoul National University Hospital, Seoul 03080, Republic of Korea; 3 CONNECTEVE Co., Ltd., Seoul 06224, Republic of Korea; 4 Innovative Medical Technology Research Institute, Seoul National University Hospital, Seoul 03122, Republic of Korea; 5 Department of Orthopedic Surgery, Samsung Medical Center, Seoul 06351, Republic of Korea

**Keywords:** lumbosacral transitional vertebrae, deep learning, artificial intelligence, automated detection, plain radiograph, convolutional neural network, spine imaging

## Abstract

**Background/Objectives**: Lumbosacral transitional vertebra (LSTV) is a common anatomical variant, but its identification on plain radiographs is often inconsistent. This inconsistency can lead to clinical complications such as chronic low back pain, misinterpretation of spinal parameters, and an increased risk of wrong-level surgery. This study aimed to develop and validate a deep learning-based artificial intelligence (AI) model for the automated detection of LSTV on plain lumbar radiographs. **Methods**: This retrospective observational study included a total of 3116 standing lumbar lateral radiographs. The presence or absence of lumbosacral transitional vertebra (LSTV) was definitively established using whole-spine imaging, CT, or MRI. Multiple deep learning architectures, including DINOv2, CLIP (ViT-B/32), and ResNet-50, were initially evaluated for binary classification of LSTV. Among these, the ResNet-50 model with partial fine-tuning achieved the best test performance and was subsequently selected for fivefold cross-validation using the training set. Model performance was assessed using accuracy, sensitivity, specificity, and the area under the receiver operating characteristic curve (AUROC), and interpretability was evaluated using gradient-weighted class activation mapping (Grad-CAM). **Results**: On the independent test set of 313 radiographs, the final model demonstrated robust diagnostic performance. It achieved an accuracy of 76.4%, a sensitivity of 85.1%, a specificity of 61.9%, and an AUC of 0.84. The model correctly identified 166 out of 195 LSTV cases and 73 out of 118 normal cases. **Conclusions**: This AI-based system offers a highly accurate and reliable method for the automated detection of LSTV on plain radiographs. It shows strong potential as a clinical decision-support tool to reduce diagnostic errors, improve pre-operative planning, and enhance patient safety.

## 1. Introduction

Lumbosacral transitional vertebra (LSTV) is a common congenital anomaly, with a reported prevalence ranging from 4% to 37% depending on the study population [1,2,3]. The clinical significance of this anatomical variant extends from its association with chronic low back pain and accelerated adjacent segment degeneration [4,5,6] to the considerable surgical challenges it presents in surgical planning. These include an increased risk of wrong-level surgery and the misinterpretation of essential spinopelvic parameters [7]. However, diagnosis based solely on plain lumbar radiographs is often inconsistent [8], as accurate vertebral enumeration typically requires whole-spine imaging, computed tomography (CT), or magnetic resonance imaging (MRI) [9].

Artificial intelligence (AI), particularly deep learning, has shown substantial promise in enhancing medical image analysis. Recently, there has been a growing trend of using foundation models trained on large image datasets. Researchers are leveraging these models by fine-tuning those initially trained on natural images with medical images such as X-rays [10,11]. In spine imaging, deep learning models have been successfully applied to tasks such as classifying spinal pathologies and automating the measurement of spinopelvic parameters [12,13]. For instance, recent studies have demonstrated the use of convolutional neural networks (CNNs) to distinguish between pathologies such as carcinoma and infection with high accuracy [12]. Other models have automated the measurement of key deformity parameters, including pelvic tilt and lumbar lordosis, with a level of consistency that may surpass that of human experts [13]. Importantly, deep learning’s ability to detect subtle imaging patterns beyond human perception offers a promising avenue for more objective and reproducible spinal diagnostics.

Despite these advances, a significant diagnostic gap remains in the automated detection of LSTV on plain radiographs. A recent deep learning–based method for identifying the L5 vertebra on lumbar spine radiographs represented a step forward in achieving accurate vertebral numbering and preventing wrong-level surgery in patients with transitional anatomy [14]. However, no prior study has specifically focused on the automated classification of LSTV itself. Therefore, this study aimed to develop and validate a deep learning model dedicated to this task. We hypothesized that the proposed model could achieve high diagnostic accuracy, providing a reliable and objective solution to the longstanding inconsistencies of manual radiographic interpretation.

## 2. Materials and Methods

### 2.1. Study Design and Dataset

**Figure 1:**
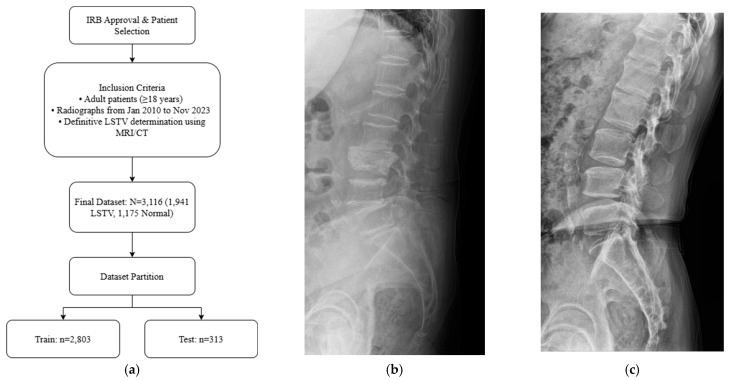
(**a**) Flow diagram of patient selection and dataset partitioning. Adult patients (≥18 years) with standing lumbar radiographs and MRI/CT-confirmed LSTV were included (*n* = 3116; 1941 LSTV, 1175 normal) and divided into training (*n* = 2803) and independent test (*n* = 313) sets. (**b**) Representative lateral radiograph showing LSTV with lumbarization of the S1 vertebra and morphological alteration of the L5–S1 junction. (**c**) Representative radiograph of a normal lumbar spine illustrating a well-defined lumbosacral junction and distinct separation between the L5 vertebra and sacrum.

Adult patients (≥18 years) who underwent standing lumbar lateral radiography at a single tertiary hospital between January 2010 and November 2023 were included for this retrospective study. All standing lumbar lateral radiographs were acquired using digital radiography systems from multiple manufacturers, including Philips Healthcare (Amsterdam, The Netherlands), Canon Medical Systems Corporation (Ōtawara, Tochigi, Japan), Samsung Electronics Co., Ltd. (Suwon, Republic of Korea), and Fujifilm Corporation (Tokyo, Japan). To establish a definitive ground truth, only patients for whom the presence or absence of LSTV could be unequivocally determined using whole-spine MRI, CT, or radiographs that allowed for accurate vertebral enumeration were selected. The presence or absence of LSTV was labeled based on the MRI conclusion that explicitly mentioned transitional vertebra, lumbarized, sacralized, lumbarization, or sacralization. Patients were excluded if they had a history of lumbar surgery with instrumentation, severe spinal deformities or pathologies obscuring local anatomy, or poor image quality (Figure 1a). The final dataset was stratified, with 10% reserved as an independent test set and the remaining 90% subjected to stratified 5-fold cross-validation for model development and validation. Representative examples of each class are shown in Figure 1b,c, illustrating the visual differences between LSTV cases and normal lumbosacral anatomy. These examples highlight the morphological variability that makes manual identification challenging.

### 2.2. Image Preprocessing and Model Development

**Figure 2:**
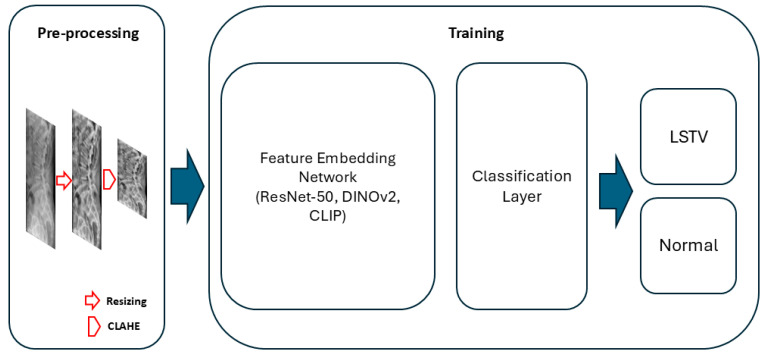
Overview of the proposed deep learning pipeline for LSTV classification. During preprocessing, lumbar spine radiographs were resized to 224 × 224 pixels and contrast-enhanced using contrast-limited adaptive histogram equalization (CLAHE). The processed images were then encoded by a feature embedding network, followed by a classification layer performing binary classification to predict LSTV or normal anatomy.

Three deep learning architectures were evaluated for model development: DINOv2-base, CLIP (ViT-B/32), and ResNet-50. For the DINOv2 and CLIP models, the vision encoder was frozen, and only the classification head—consisting of a single hidden layer (512 units) and a final output layer—was trained. For the ResNet-50 model, the first two convolutional blocks were frozen, and the remaining layers were fine-tuned. All models were trained with label smoothing (0.1) and dropout regularization (0.3–0.4). Input radiographs were resized to 224 × 224 pixels, normalized using ImageNet mean and standard deviation, and augmented through random rotation, horizontal flipping, and contrast-limited adaptive histogram equalization (CLAHE) (Figure 2).

### 2.3. Model Training and Validation

The DINOv2-base and CLIP (ViT-B/32) models were trained using the AdamW optimizer with an initial learning rate of 1 × 10^−3^, batch size of 32, weight decay of 0.01, dropout rate of 0.3, and cosine annealing learning rate scheduling over 50 epochs. The ResNet-50 model was trained using the AdamW optimizer with an initial learning rate of 5 × 10^−6^, batch size of 64, dropout rate of 0.4, weight decay of 0.01, and cosine annealing scheduling with a 5-epoch warm-up over 200 epochs.

Model performance was assessed on the test set using standard classification metrics: accuracy, sensitivity, specificity, precision, F1-score, and the area under the receiver operating characteristic curve (AUC). To compare the diagnostic performance of the AI model with human expertise, a board-certified orthopedic surgeon independently reviewed all test radiographs and classified each case as either normal or LSTV, blinded to the model outputs. The expert’s diagnostic metrics were computed using the same criteria, and direct comparisons between the AI model and the expert were made using identical evaluation measures. The best-performing model—defined as the one with the highest accuracy—was then retrained using a fivefold stratified cross-validation approach to confirm robustness and generalizability. All experiments were conducted using the PyTorch (v2.1.0) framework on an NVIDIA RTX 4060 GPU.

### 2.4. Model Interpretability

To enhance interpretability, misclassified cases were qualitatively analyzed, and gradient-weighted class activation mapping (Grad-CAM) was used to generate heatmaps. These visualizations highlighted the anatomical regions that most strongly influenced the model’s predictions.

## 3. Results

### 3.1. Dataset Characteristics

After applying the inclusion and exclusion criteria, the final dataset comprised 3116 standing lumbar lateral radiographs. Of these, 1941 (62.3%) were diagnosed with LSTV and 1175 (37.7%) were classified as normal. The dataset was divided into a training set (*n* = 2803) and an independent test set (*n* = 313). There were no significant demographic differences between the two sets.

### 3.2. Model Performance on the Test Set

**Figure 3:**
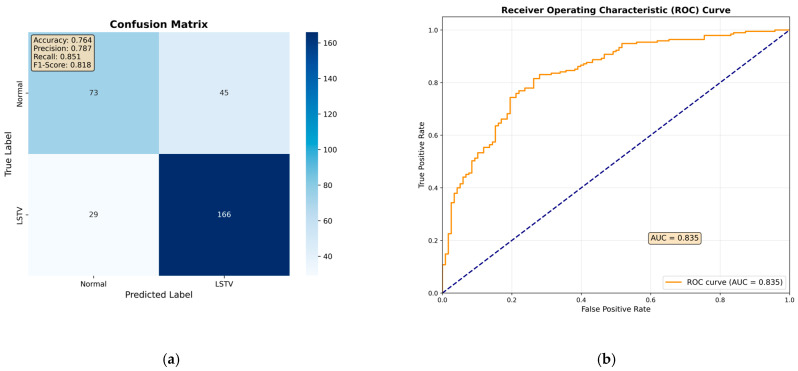
Results of the ResNet-50 model on the independent test set. (**a**) Confusion matrix showing 73 true-normal and 166 true-LSTV cases correctly classified, with 29 false negatives (LSTV misclassified as normal) and 45 false positives (normal misclassified as LSTV). (**b**) Receiver operating characteristic (ROC) curve demonstrating an area under the curve (AUC) of 0.84, indicating strong separation between LSTV and normal cases.

Among all evaluated architectures, the fine-tuned ResNet-50 model demonstrated the best overall performance on the test set, although its diagnostic accuracy did not fully match that of the orthopedic specialist (Table 1). The confusion matrix showed that the ResNet-50 model correctly identified 166 out of 195 LSTV cases and 73 out of 118 normal cases. (Figure 3a). Based on these results, the model achieved an overall accuracy of 76.4%, a sensitivity of 85.1%, a specificity of 61.9%, a precision of 78.7%, and an F1-score of 81.8% (Table 1). Furthermore, its discriminative ability was comparable to that of the orthopedic specialist, with an area under the receiver operating characteristic curve (AUROC) of 0.84 (Figure 3b).

### 3.3. 5-Fold Cross-Validation

**Figure 4:**
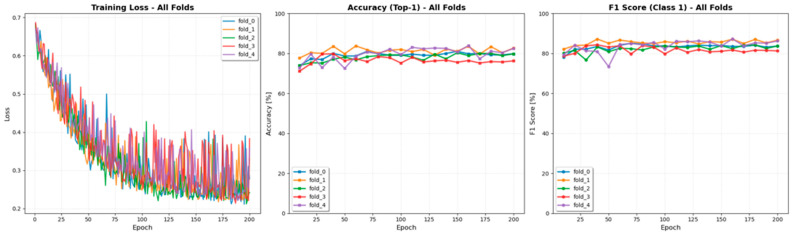
Training curves of the fivefold cross-validation from the ResNet-50 model. The plots show the iteration-wise changes in accuracy, F1-score, and training loss across folds. All folds demonstrated stable convergence and consistent performance trends, confirming the robustness of the model training process.

The ResNet-50 model, which achieved the highest accuracy among all tested architectures, was selected for cross-validation experiments. The 5-fold cross-validation on the training set confirmed the model’s stability and robustness. Performance remained consistent across all five folds, with a mean accuracy of 81.8% ± 1.9% and a mean f1-score of 85.5% ± 1.5%, indicating stable training without significant overfitting. The fold-wise variations in accuracy, F1-score, and training loss are illustrated in Figure 4.

### 3.4. Analysis of Misclassified Cases and Model Interpretability

**Figure 5:**
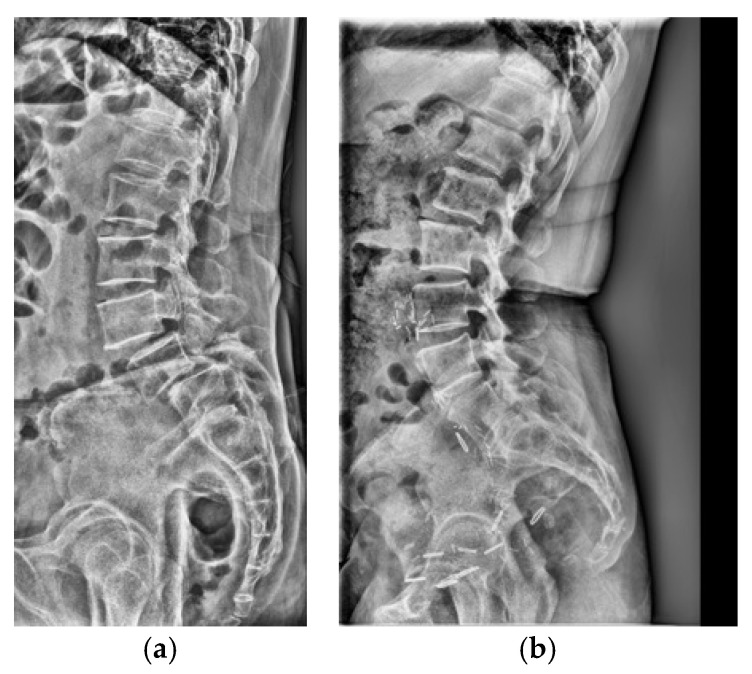
Representative misclassified cases. (**a**) Normal case misclassified as LSTV (false positive), showing subtle morphological features mimicking transitional anatomy. (**b**) LSTV case misclassified as normal (false negative), displaying transitional features similar to those in panel (**a**), which contributed to the classification difficulty on lateral radiographs.

A qualitative analysis of misclassified cases in the test set was conducted. Within the normal group, several cases exhibited features resembling LSTV, making the distinction challenging. In some instances, these cases were nearly indistinguishable from those in the LSTV group (Figure 5a). Additionally, cases involving spondylolisthesis or L5–S1 disc space narrowing were occasionally misclassified as false positives. Conversely, false negatives often showed subtle transitional characteristics or minimal spatulation, which limited definitive classification based solely on lateral radiographs (Figure 5b). These borderline presentations likely contributed to the reduced specificity and increased false-negative rate.

**Figure 6:**
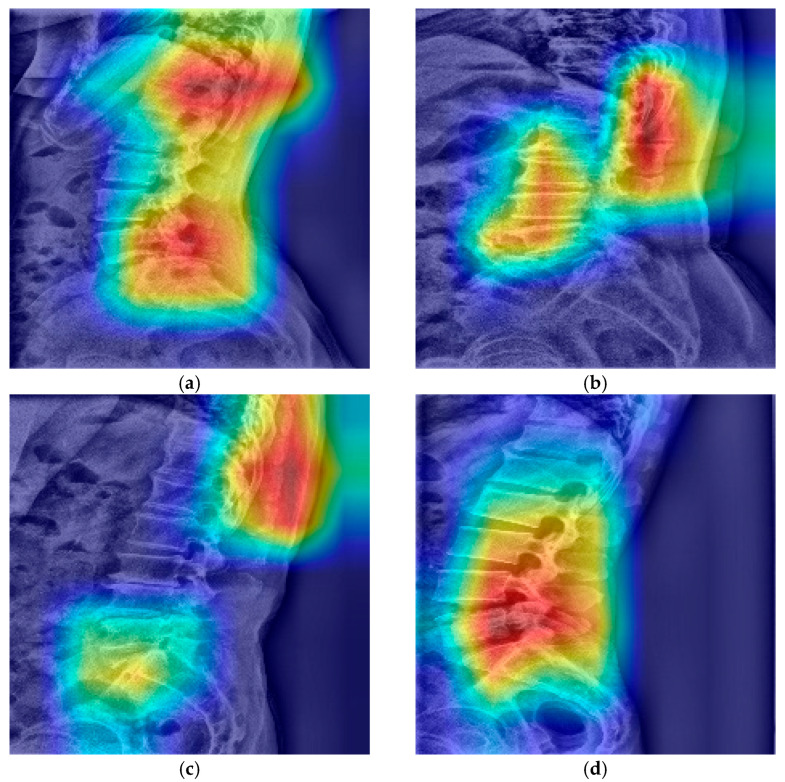
Grad-CAM visualizations illustrating the interpretability of the LSTV classifier. (**a**) Representative correctly classified LSTV case showing model attention focused on the T12 rib and L5–S1 disc space, consistent with clinically relevant anatomical cues. (**b**) Another LSTV case where the model primarily attended to the lumbar vertebral alignment, suggesting reliance on overall spinal contour. (**c**) Representative correctly classified normal case demonstrating focused attention on the T12 rib and L5–S1 disc space, similar to expert interpretation. (**d**) Normal case in which the model concentrated on the lumbar vertebral alignment rather than the lumbosacral junction, indicating variable attention patterns across normal samples.

The gradient-weighted class activation mapping (Grad-CAM) visualizations provided insight into the model’s decision-making process. For correctly classified cases, the model consistently focused on clinically relevant regions, particularly the lumbosacral junction including vertebral alignment, the L5–S1 disc space, and the T12 rib. This suggests that the model learned to recognize anatomical features similar to those used by human experts (Figure 6).

## 4. Discussion

This study developed a deep learning model for the automated detection of lumbosacral transitional vertebrae (LSTV) on plain radiographs, achieving high diagnostic performance. The model demonstrated an AUC of 0.84, accuracy of 76.4%, sensitivity of 85.1%, and specificity of 61.9%, underscoring its potential to automate a key step in diagnosis and preoperative planning. These results indicate that deep learning can effectively recognize the subtle and heterogeneous morphological features of LSTV that often hinder manual interpretation. Although the model’s accuracy was slightly below that of the orthopedic specialist, its comparable discriminative ability suggests that AI-based systems may serve as reliable assistive tools in clinical practice. Interestingly, the DINOv2-based model exhibited higher specificity than the ResNet-50 model, suggesting its potential role as a screening tool to reduce false-positive findings.

### 4.1. Challenges in Radiographic LSTV Detection

The accurate identification of LSTV on plain radiographs is a well-documented diagnostic challenge [8,15]. This limitation has prompted numerous efforts to define reliable anatomical landmarks for vertebral numbering [16,17]. However, these landmarks have proven inconsistent, particularly in transitional anatomy. For instance, Farshad-Amacker et al. reported that the iliolumbar ligament (ILL)—traditionally considered a key marker of the L5 vertebra—was unreliable in LSTV patients, originating solely from L5 in only 25–38% of cases compared with 95% in controls [16]. The same group later demonstrated that other commonly used paraspinal landmarks, such as the aortic bifurcation and right renal artery, were also unreliable due to their positional variability [17]. Although a novel “iliac crest tangent sign” was proposed for lumbar MRI, the authors concluded that only whole-spine imaging provides a true gold standard for enumeration. This persistent reliance on variable landmarks underscores the need for automated tools capable of interpreting LSTV morphology directly from imaging data—a key objective achieved by our model. Consistent with prior observations, our dataset also contained borderline normal cases that mimicked transitional anatomy, likely contributing to the model’s lower specificity and higher false-negative rate.

### 4.2. Clinical Significance and Impact on Surgical Outcomes

The identification of LSTV has significant clinical implications that extend beyond radiographic diagnosis. LSTV, with a reported prevalence of 4–37% depending on the population [1,2,3], is linked to chronic low back pain and accelerated degeneration at the adjacent cranial level [6,18]. Failure to detect LSTV contributes directly to wrong-level surgery and poor postoperative outcomes.

For instance, Ahn et al. found that patients with LSTV undergoing L4/5 microdiscectomy experienced worse pain and functional outcomes at 12 and 24 months postoperatively compared with those without LSTV [19]. The reherniation rate was also higher (6.5% vs. 0%), emphasizing the prognostic importance of transitional anatomy. Biomechanically, Farshad-Amacker et al. demonstrated that the increased mechanical connection in Castellvi Types II–IV provided stability at the transitional level but increased stress and degeneration at the superior adjacent segment [20].

The implications extend to deformity surgery. Lee et al. suggested that the presence of LSTV should influence distal fusion level selection in adolescent idiopathic scoliosis (AIS); for example, stopping at L3 rather than L4 may preserve motion and protect the L4–5 disc [21]. Similarly, Ani et al. reported that in adult spinal deformity, sacralized L5 leads to the underestimation of deformity when spinopelvic parameters are measured from L5 instead of S1 [7]. Thus, accurate detection of LSTV is essential for optimal surgical planning and risk stratification.

Our model’s ability to identify subtle LSTV morphology directly from radiographs may reduce diagnostic subjectivity and enhance surgical safety. By offering a consistent, objective detection method, it also addresses the wide variability in reported LSTV prevalence (4–37%) caused by inconsistent diagnostic criteria [1,2,3].

### 4.3. Clinical Applications and Future Directions

The proposed model can be integrated as an automated “second reader” to assist clinicians in identifying LSTV, particularly during preoperative planning. By flagging potential cases for further review, the system may help prevent wrong-level surgeries and improve diagnostic accuracy. A practical workflow could involve follow-up whole-spine imaging for AI-flagged cases, thereby enhancing efficiency while minimizing unnecessary scans.

The model’s strong sensitivity (85.1%) is its primary clinical advantage, ensuring that few true LSTV cases are missed. However, the lower specificity (61.9%) indicates a higher likelihood of false positives. To maintain resource efficiency, the model should serve as a high-yield screening and triage tool rather than a definitive diagnostic instrument. A cost-effective clinical workflow would involve using the AI flag to prompt targeted confirmatory imaging; follow-up whole-spine or cross-sectional imaging (CT/MRI) would be warranted only for AI-flagged cases. This approach improves the overall diagnostic accuracy and efficiency by focusing resources on the most suspicious cases, thus reducing unnecessary advanced imaging for the general population.

Additionally, this model may serve as a foundational module for comprehensive spine imaging systems, ensuring accurate landmark localization (e.g., the true S1 endplate) for subsequent AI-based parameter measurements.

Future studies should expand the model’s capabilities to include automated Castellvi sub-classification (Types I–IV), which carries distinct clinical implications. Incorporating expert-defined regions of interest (ROIs) into training could further improve model focus and interpretability, aligning its learning with human diagnostic reasoning.

### 4.4. Limitations

This study has several limitations. First, the model was developed using data from a single institution, and its generalizability across different populations and imaging protocols remains to be validated. Multi-center external validation is needed to confirm robustness. Second, the model currently performs only binary classification (presence/absence of LSTV) and does not provide Castellvi subtypes, which are clinically relevant. Future versions should address this limitation. Third, the retrospective design is subject to selection bias and may not capture the full spectrum of image quality encountered in daily practice. In particular, the dataset may underrepresent the challenges associated with low-quality radiographs, such as those frequently obtained from obese or morbidly obese patients, who are common in Western populations. A prospective study in a real-world clinical workflow is needed to validate the model’s performance under such variable conditions. Fourth, while the model achieved strong diagnostic performance (AUC = 0.84), it did not surpass the orthopedic specialist. Nevertheless, its near-expert performance demonstrates its potential as a reliable assistive tool for screening and triage. Finally, interpretability remains a challenge due to the model’s “black-box” nature. Incorporating vision language models capable of providing transparent reasoning and visual–textual explanations may help address this limitation.

## 5. Conclusions

In conclusion, we successfully developed a deep learning model capable of accurately automating the detection of LSTV on plain radiographs. This model addresses a critical clinical need by providing a consistent and objective solution to a challenging diagnostic problem, thereby improving patient safety and supporting surgical planning. Although further external validation and functional expansion are required, this study represents a meaningful step toward integrating advanced AI tools into routine spine care.

## Figures and Tables

**Table 1:** Comparison of classification performance among three AI models and an orthopedic specialist for detecting LSTV on plain radiographs. Accuracy, sensitivity, specificity, precision, F1-score, and AUC are reported for each model and the human expert.

	Accuracy (%)	Sensitivity (%)	Specificity (%)	Precision (%)	F1-Score (%)	AUC
DINOv2 + FC	75.4	90.8	50.0	75.0	82.1	0.80
CLIP + FC	71.0	79.5	56.8	75.2	77.3	0.73
ResNet-50	76.4	85.1	61.9	78.7	81.8	0.84
Orthopedist	83.9	81.3	87.3	91.3	85.9	

## Data Availability

Data underlying this article cannot be shared publicly due to the privacy of the individuals who participated in this study. The data may be shared upon a reasonable request by the corresponding author.

## Abbreviations

The following abbreviations are used in this manuscript:

LSTV	Lumbosacral transitional vertebra
AUROC	Area under the receiver operating characteristic curve
AI	Artificial intelligence
Grad-CAM	Gradient-weighted class activation mapping
CT	Computed tomography
MRI	Magnetic resonance imaging
CNNs	Convolutional neural networks
CLAHE	Contrast limited adaptive histogram equalization
